
JOURNAL INFORMATION
==============================
NLM Title Abbreviation: ScientificWorldJournal
Journal ID: ScientificWorldJournal
NLM Journal ID: 101131163
Journal ID: TSWJ

The Scientific World Journal

ISSN: 2356-6140
EISSN: 1537-744X
Publisher: Wiley

ARTICLE INFORMATION
==============================
PMCID: PMC6084215
DOI: 10.1100/tsw.2001.416
Article ID: 910782
Article version: 1
Subjects: Extended Abstract

A Novel Nonpeptide CGRP Receptor Antagonist, ‘Compound 1’ (WO98/11128) Is a High Affinity Competitive Antagonist at the CGRP1 Receptor in Human Coronary Arteries

Hasbak P. 1 2
Opgaard O. Saetrum 3
Eskesen K. 1
Schifter S. 2
Arendrup H. 4
Longmore J. 5
Edvinsson L. 1
1 Department of Clinical Experimental Research, University Hospital of Copenhagen, Glostrup, Denmark
2 Department of Clinical Physiology and Nuclear Medicine, University Hospital of Copenhagen, Glostrup, Denmark
3 Department of Pharmacology, College of Medicine, University of California at Irvine, Irvine, CA, USA
4 Department of Thoracic Surgery, Rigshospitalet, Copenhagen, Denmark
5 Merck Sharp & Dohme, Neuroscience Research Centre, Harlow, Essex, UK
Publication date: 2001
Electronic publication date: 2001 Jun 15
Volume: 1
First page: 33
Last page: 33
Copyright: Copyright © 2001 P. Hasbak et al.
Copyright year: 2001
License: This is an open access article distributed under the Creative Commons Attribution License, which permits unrestricted use, distribution, and reproduction in any medium, provided the original work is properly cited.
License URL: https://creativecommons.org/licenses/by/3.0/

==============================
4th International Meeting on Calcitonin Gene-Related Peptide (CGRP)
*TheScientificWorld* (2001) 1(S1), 33
ISSN 1532-2246; DOI 10.1100/tsw.2001.416

### A NOVEL NONPEPTIDE CGRP RECEPTOR ANTAGONIST, 'COMPOUND 1' (WO98/11128) IS A HIGH AFFINITY COMPETITIVE ANTAGONIST AT THE $CGRP_1$ RECEPTOR IN HUMAN CORONARY ARTERIES

P. Hasbak[a,b], O. Saetrum Opgaard[c], K. Eskesen[a], S. Schifter[b], H. Arendrup[d], J. Longmore[e], and L. Edvinsson[a]

[a]Department of Clinical Experimental Research, University Hospital of Copenhagen, Glostrup, Denmark; [b]Department of Clinical Physiology and Nuclear Medicine, University Hospital of Copenhagen, Glostrup, Denmark; [c]Department of Pharmacology, College of Medicine, University of California at Irvine, Irvine, CA; [d]Department of Thoracic Surgery, Rigshospitalet, Copenhagen, Denmark; [e]Merck Sharp & Dohme, Neuroscience Research Centre, Harlow, Essex, U.K.

Functional CGRP and adrenomedullin receptors appear to consist of at least three different kinds of proteins: the calcitonin receptor-like receptor (CRLR), receptor-activity-modifying protein (RAMPs), and the receptor component protein (RCP) that couples the receptor to the cellular signal transduction pathway.

Using reverse transcriptase - polymerase chain reaction the presence of mRNA sequences encoding CRLR, RCP, and the three types of human RAMP that have been cloned were demonstrated in human left anterior descending (LAD) coronary arteries.

Relaxant responses were studied on isolated segments of LAD after precontraction with U46619 ($10^{-7}$ M). The human peptides $\alpha$CGRP, AM, and amylin induced relaxation with $pEC_{50}$ values of $8.2 \pm 0.2$, $6.9 \pm 0.1$, and $6.3 \pm 0.2$ M (mean $\pm$ S.E.M.), respectively.

The antagonistic properties of a novel nonpeptide CGRP antagonist 'Compound 1' (WO98/11128) were compared to the well-known $CGRP_1$ receptor antagonist $\alpha CGRP_{8-37.}$ Preincubation with $\alpha CGRP_{8-37}$ ($10^{-7}$ to $10^{-5}$ M) and 'Compound 1' ($10^{-7}$ to $10^{-5}$ M) caused a dose-dependent rightward shift of the concentration-response curves for $\alpha$CGRP. The $pA_2$ values were 7.3 and 7.0,respectively.

Preincubation with $\alpha CGRP_{8-37}$ ($10^{-6}$ M) and 'Compound 1' ($10^{-6}$ M) caused significant rightward shift of the concentration-response curves for AM and amylin. Preincubation with $AM_{22-52}$ ($10^{-6}$ M) had no significant antagonistic effect.

In conclusion, mRNA for the components forming CGRP and AM receptors were present in the human LAD. $\alpha$CGRP, AM, and amylin mediated vasorelaxation via the $CGRP_1$ receptor. Compound 1 acted as a selective nonpeptide antagonist at the CGRP receptor and could thus become a tool for the study of CGRP-mediated functional responses in human tissue.
